# Identification and validation of mutation points associated with waxy phenotype in cassava

**DOI:** 10.1186/s12870-020-02379-3

**Published:** 2020-04-15

**Authors:** Cátia Dias do Carmo, Massaine Bandeira e Sousa, Priscila Patrícia dos Santos Silva, Gilmara Alvarenga Fachardo Oliveira, Hernán Ceballos, Eder Jorge de Oliveira

**Affiliations:** 1 Universidade Federal do Recôncavo da Bahia, Campus Cruz das Almas, CEP, Cruz das Almas, BA 44380-000 Brazil; 2 International Center for Tropical Agriculture (CIAT), A.A 6713 Cali, Colombia; 3 Embrapa Mandioca e Fruticultura, Rua da Embrapa, Caixa Postal 007, CEP, Cruz das Almas, BA 44380-000 Brazil

**Keywords:** Waxy starch, Screening, Sequencing, KASP, Breeding

## Abstract

**Background:**

The granule-bound starch synthase I (GBSSI) enzyme is responsible for the synthesis of amylose, and therefore, its absence results in individuals with a waxy starch phenotype in various amylaceous crops. The validation of mutation points previously associated with the waxy starch phenotype in cassava, as well as the identification of alternative mutant alleles in the GBSSI gene, can allow the development of molecular-assisted selection to introgress the waxy starch mutation into cassava breeding populations.

**Results:**

A waxy cassava allele has been identified previously, associated with several SNPs. A particular SNP (intron 11) was used to develop SNAP markers for screening heterozygote types in cassava germplasm. Although the molecular segregation corresponds to the expected segregation at 3:1 ratio (dominant gene for the presence of amylose), the homozygotes containing the SNP associated with the waxy mutation did not show waxy phenotypes. To identify more markers, we sequenced the GBSS gene from 89 genotypes, including some that were segregated from a cross with a line carrying the known waxy allele. As a result, 17 mutations in the GBSSI gene were identified, in which only the deletion in exon 6 (MeWxEx6-del-C) was correlated with the waxy phenotype. The evaluation of mutation points by discriminant analysis of principal component analysis (DAPC) also did not completely discriminate the waxy individuals. Therefore, we developed Kompetitive Allele Specific PCR (KASP) markers that allowed discrimination between WX and wx alleles. The results demonstrated the non-existence of heterozygous individuals of the MeWxEx6-del-C deletion in the analyzed germplasm. Therefore, the deletion MeWxEx6-del-C should not be used for assisted selection in genetic backgrounds different from the original source of waxy starch. Also, the alternative SNPs identified in this study were not associated with the waxy phenotype when compared to a panel of accessions with high genetic diversity.

**Conclusion:**

Although the GBSSI gene can exhibit several mutations in cassava, only the deletion in exon 6 (MeWxEx6-del-C) was correlated with the waxy phenotype in the original AM206–5 source.

## Background

Starch is widely used for multiple commercial purposes. Not only does it provide more than 80% of the calories in the human diet [1], it also has many industrial applications, such as for glues and adhesives. It basically consists of two types of polymers: amylose (essentially α1,4-polyglucans, linear) and amylopectin (α1,4-polyglucans and α-1,6-polyglucans, branched) [2], whose proportions and arrangements confer characteristics that define the starch’s commercial advantages and are the focus of breeding programs. The main commercial sources of starch are maize, cassava, rice, wheat and potato. Cassava is the second most important source of starch worldwide after maize. The functional and specific properties of each of these species define their industrial applications [3–6].

Amylopectin, the basic unit of the starch granule, is composed of many short chains of glucose molecules. In contrast, amylose is a smaller molecule with longer chains [7]. Starch from different crops typically consists of 20–30% amylose and 70–80% amylopectin [1]. Starches with low or no amylose, known as waxy types, are important because they present lower syneresis and retrogradation, are generally clear and have a more viscoelastic gel form [8]. Retrogradation is a process in which the disrupted amylose and amylopectin chains (usually heated in the presence of water) can gradually re-associate into a different ordered structure. Over time, this reorganization can release the water retained within the structure (syneresis), with detrimental effects on the sensory qualities and storage time of foods derived from this type of starch, primarily because it alters the texture and nutritional properties of the food [9]. Not only does waxy cassava starch present lower syneresis in comparison with waxy starch from other crops [10], the structure of amylopectin is not modified by the mutation [11]. Cassava starch has a neutral taste due to its low contents of lipids and proteins, which gives it competitive advantages over starches from cereals for use in the food industry [12].

Though it is possible to perform chemical and physical modifications of starch to change its functional properties to meet specific market demands, there are certain limitations on these modifications. In addition, regardless of the type, making such changes to starch is always associated with a higher industrial cost. Another aspect to be considered is that consumers are increasingly demanding products that are more natural, with a minimum of industrial processing. Hence, there is a growing market for naturally differentiated starches. Breeding programs can contribute to the discovery of genetic variants of interest and the further incorporation of these characteristics in commercial varieties, and waxy starch is one of these special traits.

From the genetic point of view, a clear understanding of the genes involved in the starch biosynthesis allows the identification of accessions from germplasm carrying the mutations without the need for a laborious phenotyping approach. Among the enzymes related to starch synthesis, GBSSI (granule-bound starch synthase I) is a synthase related to amylose elongation. Waxy mutants of many species exhibit deficient activity of this enzyme [7, 13, 14]. Several allelic forms derived from different molecular mechanisms have been identified as responsible for the waxy phenotype. In maize, the coding region of the waxy gene comprises 3718 bp, which is composed of 14 exons, ranging from 64 to 392 base pairs (bp), and 13 introns of 81 to 139 bp [15]. According to Fan et al. [16] the *wx*-D7 allele involves a 30-bp deletion at the junction of the 7th exon–intron and generates an abnormal transcript retaining the 7th intron, which introduces an immature stop codon and inactivates GBSSI. In rice, two SNPs were identified in exons 6 and 10 of the GBSSI gene, with associations to the amylose content and paste properties [17]. A strong correlation was also observed between an SNP located at exon1/intron1 and paste properties in rice starch [18]. In common hexaploid wheat (*Triticum aestivum* L.), each of the genomes A, B and D has a waxy protein, whose respective locus coder (Wx-A1, Wx-B1, and Wx-D1) has several mutations reported in the literature. In another work, the insertion of an SNP and deletion of a single nucleotide in the null allele Wx-A1 induced premature termination codons approximately 55 nucleotides forward of the mutation point in *Triticum dicoccoides* A*.* and *T. dicoccum* S [19]. More recently, a new null allele was characterized by the insertion of a transposon and consequent loss of function in this species [20]. Two new mutations have also recently been discovered in maize, associated with transposable elements, with a 466-bp retrotransposon inserted in exon 6 and a transposable repeating element of 116 bp inserted in exon 7 [21].

In cassava, studies of the genetic control of the waxy phenotype and even its exploitation for the development of varieties with this characteristic are relatively recent. The first waxy clones in cassava were induced by modifications in GBSSI gene expression via transgenic techniques [22, 23] and later by Zhao et al. [24]. Then, a natural mutation was reported by [25] in a series of self-pollinations carried out at the International Center for Tropical Agriculture (CIAT), which evidenced the recessive nature of the waxy gene from genotype AM206–5. Recently, the CRISPR-Cas9 technology was used in cassava to mediate targeted mutagenesis of two genes involved in amylose biosynthesis (GBSSI and Protein Targeting to Starch - PTST1) resulting in waxy clones or clones with reduced amylose content [26].

Aiemnaka et al. [27] performed molecular characterization of the GBSSI gene in segregating progenies from the AM206–5 source and identified potential functional mutations. The authors found an indel in exon 6 with a single base exclusion (cytosine) that creates a premature stop codon; a two-base variant in exon 11 (GC and AT); and a substitution in intron 11 (C to G). Based on this last mutation, the authors developed a SNAP (single-nucleotide-amplified polymorphism) marker whose information would allow breeders to direct self-pollination and backcrossing assisted by molecular markers, with the aim of reducing the costs and time required for breeding programs. In this context, the objectives of the present work were as follows: i) to validate the mutations (SNPs and indels) previously associated with the original source of the waxy starch from AM206–5 in Latin American cassava germplasm; and ii) to identify alternative alleles in the GBSSI gene in a panel of waxy and non-waxy accessions that may be useful in molecular-assisted selection (MAS) for this trait.

## Results

### Screening of cassava germplasm with SNAP markers

SNAP primers developed previously for intron 11 [27] were used to screen 1529 cassava accessions from four countries. The profile analysis of the electrophoresis gels was based on the presence or absence of the fragment amplified by the primers MeWxI11-G and MeWxI11-C, which indicated the presence of heterozygous individuals, whereas the amplification of only one of the two alleles indicated the presence of homozygous individuals for the waxy (MeWxI11-G - GG genotype) or non-waxy (MeWxI11-C, CC genotype) phenotype. According to the molecular analyses, none of the cassava accessions evaluated were identified as having the recessive allelic condition (GG). This result was expected, since none of the 1529 genotyped accessions had the waxy starch phenotype. However, 206 of the evaluated individuals were identified as heterozygotes (CG), because they had alleles amplified by the primers MeWxI11-C and MeWxI11-G.

### Genotypic and phenotypic evaluation of S_1_ populations with SNAP markers

Twenty-eight heterozygous accessions from the total (206) were self-pollinated in the field with the aim of generating S_1_ segregation populations for the waxy gene. From this total (28), three heterozygous accessions (BGM0061, BGM0935 and BGM0438) produced 187 plants (61, 101 and 25, respectively) that were used to evaluate the molecular segregation of the alleles and the waxy phenotype. The results of the allelic segregation of the S_1_ progenies, analyzed with the primers MeWxI11-C and MeWxI11-G, showed no significant deviations from the expected proportion of individuals (1:2:1) in progenies S_1_-BGM0061, S_1_-BGM0935 and S_1_-BGM0438, according to the χ^2^ test (Table 1). Therefore, even with a small number of individuals in these three progenies, it was possible to observe the Mendelian segregation of the C and G alleles located at intron 11, position 3197 bp of the GBSSI gene, as expected for a recessive trait (Supplement Fig. S1). However, although molecular segregation occurred as expected in progenies S_1_ - BGM0061, S_1_ - BGM0935 and S_1_ - BGM0438, the evaluation of the waxy phenotype in all 28 of the S_1_ progenies in the field, based on the 2% iodine test, did not identify any individual with the waxy phenotype. Therefore, this result showed that the mutation of the C by G allele at the 3197 bp position of the GBSSI gene is not a suitable molecular marker for use in molecular-assisted selection of waxy individuals when the genetic background is completely different from the original source of the mutation (AM206–5).

**Table 1:** Molecular segregation of the single nucleotide polymorphism (SNP) at position 3197 bp (Intron 11) of the granule-bound starch synthase I (GBSSI-I) gene in three cassava S_1_ progenies

S_1_ progenies	GC	CC	GG	GC + CC		GG	χ^2^
				f_o_	f_e_*	f_e_*	
S1- BGM0061	32	16	13	48	45.75	15.25	0.44
S1- BGM0935	41	38	22	79	75.75	22.25	0.56
S1- BGM0438	10	8	7	18	18.75	6.25	0.12
Total	84	60	41				

f_o_ - Frequency observed and f_e_ – Frequency expected according to Mendelian segregation 1:2:1

### Identification of new genetic variation in the GBSSI by gene sequencing

**Fig. 1:**
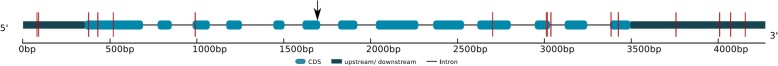
Schematic representation of the GBSSI (granule-bound starch synthase I) gene indicating the presence of single nucleotide polymorphisms (SNPs) identified in 90 cassava accessions. The red lines represent the SNPs, and the arrow indicates the deletion

**Fig. 2:**
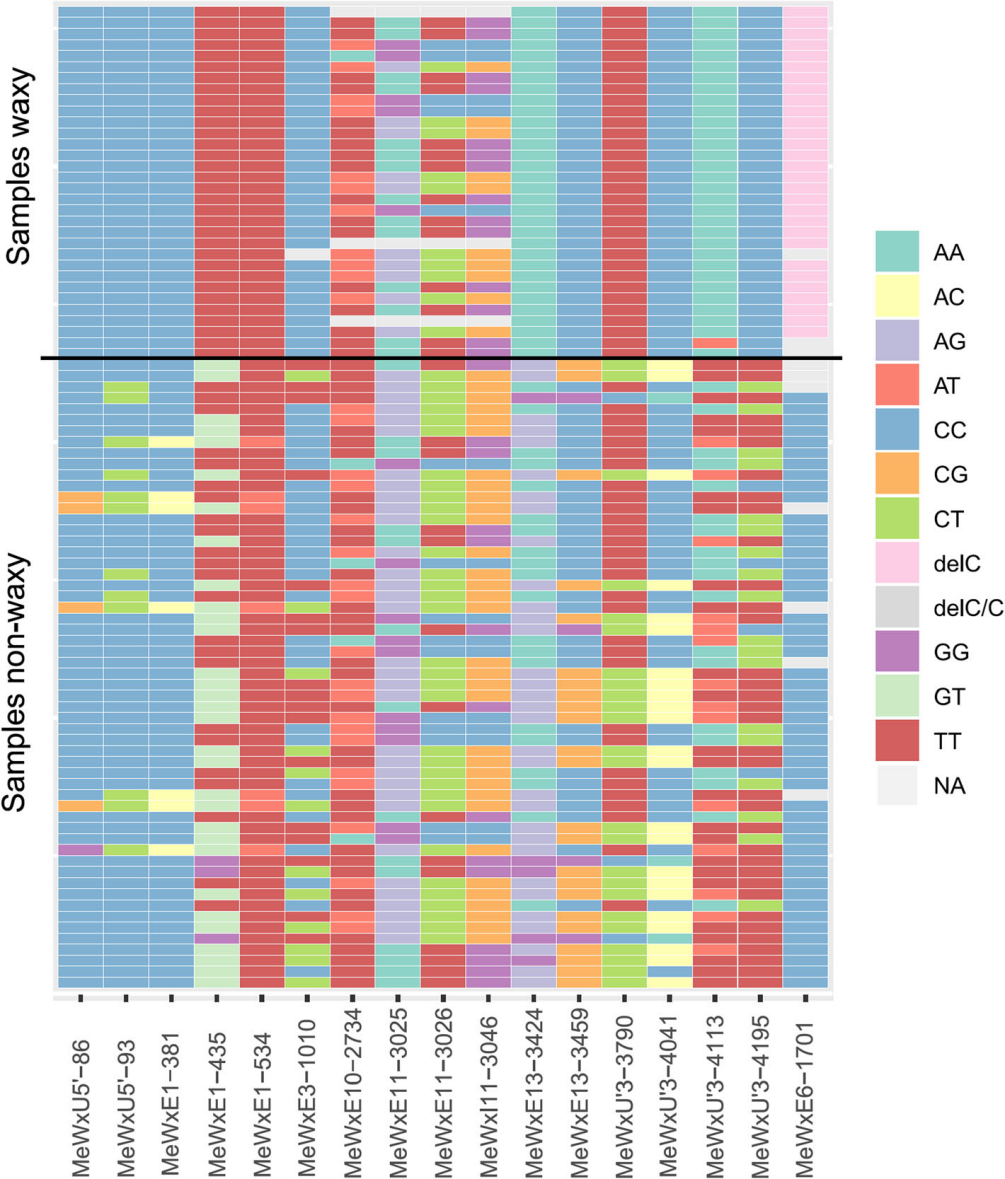
Allelic variation of single nucleotide polymorphisms (SNPs) and indels identified in the sequencing of the GBSSI gene in waxy and non-waxy cassava genotypes. Numbers forward of the SNP coding represent the position in the GBSSI gene (in base pairs). Code: Me - species (*M. esculenta*); Wx - gene (GBSSI); U’5 - UTR’5 region, E - coding regions (exon); I - non-coding regions (introns); U’3 - UTR’3 region

To identify new allelic variants associated with waxy starch, complete GBSSI gene sequencing was performed on 89 cassava genotypes. After the alignment trimming of the sequences, 16 SNPs were identified in the GBSSI gene as well as one deletion, indicating genotypic differences among the 89 cassava accessions evaluated, which included waxy (AM206–5 derived population) and non-waxy genotypes (germplasm bank) (Fig. 1). Six SNPs were found in untranslated regions (UTRs), one in a non-coding region and nine in coding regions. However, the allelic variation identified in the GBSSI gene was not able to identify any SNP that could precisely distinguish the waxy and non-waxy individuals (Fig. 2). Only the deletion of the nucleotide cytosine (MeWxEx6-del-C) previously identified by Aiemnaka et al. [27] was found exclusively in waxy accessions. Therefore, the SNPs identified in the GBSSI gene were not able to individually determine stop codons or errors in the genetic code reading that led to the development of a waxy phenotype in cassava.

### Discriminant analysis of principal components (DAPC) of the waxy and non-waxy genotypes based on the allelic variation of the GBSSI gene

Additionally, DAPC was performed to determine a discriminant function for clustering the different individuals based on the set of SNPs found in the GBSSI gene and the starch types. Only the 12 SNPs that showed minimum variation between the waxy and non-waxy individuals were used. The SNPs of exon 10 (position 2734 bp) and exon 11 (positions 3025, 3026 and 3046 bp) were the most divergent among individuals with the waxy phenotype (Fig. 2) and were withdrawn from the joint analysis. The deletion MeWxEx6-del-C (position 1701 bp) was also removed from the analysis, since the main objective was to determine how the other mutations could jointly discriminate the waxy phenotype, in order to increase the possibilities of identifying individuals with waxy alleles in the cassava germplasm from Brazil.

**Fig. 3:**
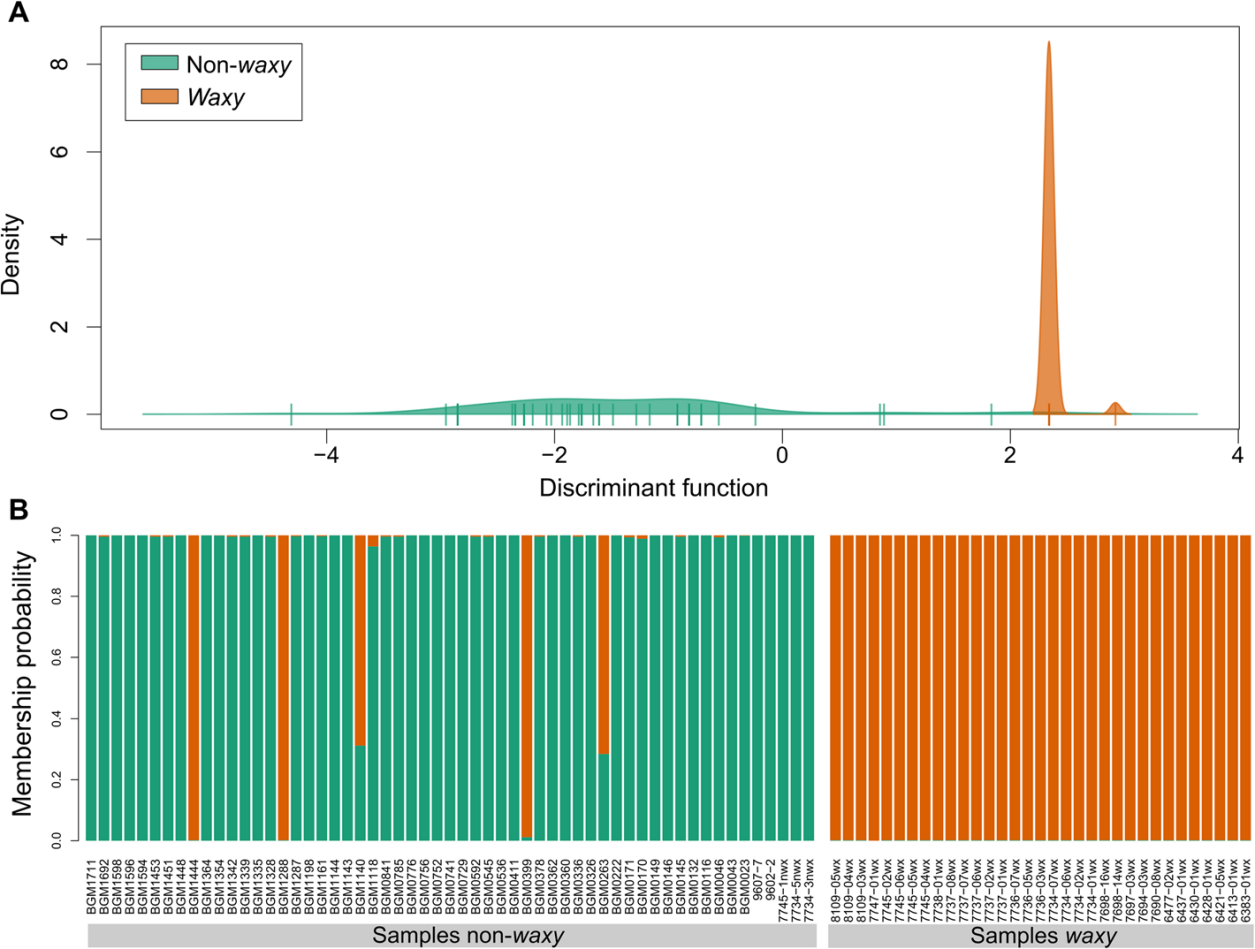
Discriminant analysis of principal components (DAPC) based on the analysis of 12 single nucleotide polymorphisms (SNPs) identified in the GBSSI gene in waxy and non-waxy starch individuals. **a** Density graph of the first discriminant functions; **b** Membership assignment of the individuals to the a priori clusters defined with DAPC

The overlap of the discriminant function density and the membership assignment indicated that the combination of SNPs was not able to cluster the waxy and non-waxy individuals with 100% accuracy (Fig. 3). Although the classification model demonstrated good accuracy, there was no complete discrimination (100%) of **non-waxy** individuals (BGM1444, BGM1288, BGM0399, BGM1140, and BGM0263), according to its membership assignment (Fig. 3).

The SNPs that contributed most to the discriminant function are located in the 3’UTR at 4041 and 4195 bp, and exon 13 at 3459 bp. Since the non-waxy individuals BGM1444, BGM1288 and BGM0399 shared these same SNPs with the waxy individuals, the waxy phenotype discrimination could not be performed accurately. The non-waxy individuals BGM1140 and BGM0263 shared the SNPs with small contributions (≤ 0.01) to discriminate the phenotype, located in the 5’UTR at 86 and 93 pb, as well as exon 1 at 381 and 534 bp and exon 3 at 1010 bp. The clone BGM1140 also shared the SNP of 3’UTR region (4195 bp) with waxy individuals.

### Genotypic evaluation of the germplasm bank via KASP

After the complete sequencing of the GBSSI gene, we also identified a deletion in exon 6 at position 1701 bp (henceforth referred to as MeWxEx6-del-C), previously described by Aiemnaka et al. [27]. Considering that only the deletion of cytosine at the 1701 bp position of the GBSSI gene was able to distinguish the waxy from non-waxy phenotypes (Fig. 2), the next step was to implement a genotyping system for this deletion on a large scale. Thus, an initial analysis with the KASP (Kompetitive Allele Specific PCR) technique was performed to evaluate the amplification of the target fragments. In the previous genotyping, the SNAP primers MeWxI11-G and MeWxI11-C were enriched with three nucleotides at the 3′ end to allow differential amplification of an SNP at intron 11. The same strategy was used in the other mutations reported by Aiemnaka et al. [27], without success [data not shown]. In contrast, KASP technology could detect only one base difference by the use of primers differentially marked by fluorescence [28].

**Fig. 4:**
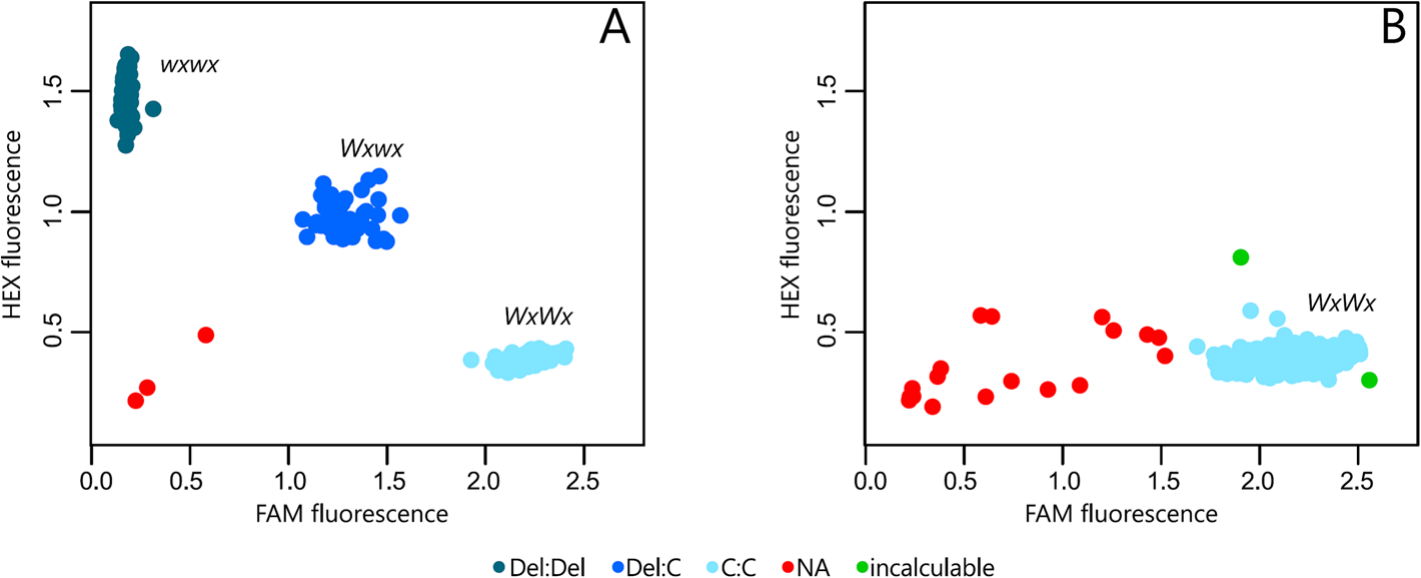
Genotyping via Kompetitive Allele Specific PCR (KASP) for the deletion MeWxE6-del-C. **a** Training population composed of 94 cassava accessions including 31 waxy starch homozygotes (*wxwx*); 31 non-waxy starch heterozygotes (*Wxwx*); and 32 non-waxy starch homozygotes (*WxWx*); and **b** Validation population composed of 1529 cassava accessions from Latin America

HEX fluorescence was used to detect waxy homozygous (*wxwx*) accessions, and FAM fluorescence to detect non-waxy homozygous (*WxWx*) accessions, while the heterozygous accessions were identified by intermediate fluorescence (*Wxwx*). In the first analysis of the MeWxEx6-del-C deletion via KASP, we used a set of 94 accessions, and only the 2017wx-02-17 and 7934–1 genotypes did not generate consistent signals for fluorescence detection or did not amplify (Fig. 4), while the genotypes 2017wx-01-02 and 2017wx-02-19 did not present the expected alleles. Therefore, this initial analysis yielded a 96% success rate in identifying the waxy and non-waxy phenotypes. Then the 1529 germplasm accessions were genotyped for validation of the method, and at this stage there were only 18 genotypes (S1–1662-65, BGM-0052, BGM-0056, BGM-0314, BGM-0336, BGM-1037, BGM-1415, BGM-1598, BGM-1698, BGM-1716, BGM-1756, BGM-2245, BGM-2252, BGM-2257, BGM-2278, BGM-0150, BGM-0856, Brasileira) for which we could not identify the alleles associated with the waxy phenotype (Fig. 4). Thus, KASP allowed the precise identification of 98% of the individuals.

The main results of these analyses were as follows: i) absence of heterozygous cassava accessions for deletion of the C allele at position 1701 bp of the GBSSI gene in the evaluated germplasm collection; and ii) high specificity in the identification of the allelic condition of the cassava genotypes. Therefore, this point mutation can allow the accurate identification of the allelic condition of the waxy gene for any cassava germplasm (Fig. 4).

## Discussion

The main objective of the initial evaluation of cassava germplasm with SNAP markers was to identify the presence of alleles associated with the waxy phenotype and then guide the self-pollination of the accessions once the waxy phenotype is expressed under the recessive condition. So, we used primers related to the waxy mutations previously described by Aiemnaka et al. [27] in the original source AM205–6. However, the inefficiency of the MeWxI11-G/C primers for the identification of individuals with the waxy phenotype in the germplasm from Latin America was demonstrated when evaluating the 28 S_1_ families derived from self-pollination identified as having the G allele associated with the waxy trait. The reported primers did not identify the waxy phenotype, since the S_1_ individuals with the G/G genotype had high amylose content according to the iodine test in the field. Therefore, it is possible to speculate that the SNP previously identified by Aiemnaka et al. [27] (C-G at position 3197 bp) can be used for molecular marker-assisted selection only in segregating populations derived from the AM205–6 source, and it is not possible to validate its use for different genetic backgrounds.

Based on the complete GBSSI gene sequencing performed in waxy and non-waxy individuals, all mutations described by Aiemnaka et al. [27], comprising one indel in exon 6 (MeWxE6-del-C), two SNPs in exon 11 (MeWxE11–3025 and 3026), and one substitution in intron 11 (MeWxI11–3046), were identified. In addition, 13 other SNPs in the GBSSI gene were also found (MeWxU’5–86 and 93; MeWxE1–381,435, 543; MeWxE10–2734; MeWxE13–3424, 3459; MeWxU’3–3790, 4041, 4113, 4195). However, out of the 16 SNPs identified in the GBSSI gene, none of them were individually able to distinguish waxy and non-waxy phenotypes.

For the GBSSI gene sequencing, a panel of 90 cassava accessions from different origins in Latin America representing the great genetic diversity of this crop was used. This high diversity was crucial to identify SNPs that were common to waxy and non-waxy genotypes. The data are therefore consistent and can serve as a basis for further investigations of cassava in relation to waxy starch. Similar results have also been reported for Indian rice varieties in Northeast India, where again mutations in the GBSSI gene previously associated with amylose content did not distinguish the waxy phenotype [29]. Of the five varieties of waxy rice evaluated by the authors, two did not have the mutation, and among the non-waxy, one mutation occurred in one variety. Choudhury et al. [29] also investigated the OsC1 locus responsible for the apiculus color in rice, and they found that a 10 bp deletion in the OsC1 gene, attributed to a colorless apiculus, was not detected in five of the 21 varieties with this phenotype. Moreover, one of the nine rice varieties with a colored apiculus presented the deletion. Thus, the mutations considered to be associated with waxy starch and apiculus color in rice did not necessarily correspond to the expected phenotype in different genetic backgrounds.

Only the deletion MeWxE6-delC (position 1701 bp) was exclusive of waxy individuals from the AM206–05 original source and thus able to characterize the waxy phenotype in these populations. This deletion creates a premature TGA stop codon at Exon 8 (position 2069 bp) that prevents complete synthesis of the GBSSI enzyme [27]. GBSS is bound to the granule, most likely by synthesizing the amylose within the granular matrix formed by amylopectin. Therefore, mutants produce less or no amylose, suggesting that no other synthase can replace it in this function [30].

The KASP genotyping for screening of the MeWxE6-del-C deletion showed high accuracy of waxy and non-waxy classification [98%]. Indeed, other authors have reported the great flexibility and high accuracy of the KASP technique for high-performance genotyping [28]. The KASP genotyping allowed a clear distinction between the homozygous and heterozygous accessions in the training population, evidencing its great potential in molecular-assisted selection. In this sense, our work is an important advance for cassava breeding, as it validates KASP genotyping for routine detection of the waxy phenotype by the marker MeWxEx6-del-C, when using alleles from the AM206–05 source. In the future, high-throughput screening (HTS) with several important functional markers for cassava will be possible, as has been the case in other crops such as wheat, for which more than 38 polymorphisms from different agronomic traits have been analyzed with the KASP technique [31]. The speed of the KASP genotyping assays was 45 times higher than that of the gel-based PCR markers.

Analyses in different species support the perception that the waxy phenotype is related to the absence/deficiency of the GBSSI enzyme, associated with partial and complete deletions of the gene, the presence of SNPs, and transposable elements, sometimes leading to stop codons [17–21, 32, 33]. In cassava, a first natural recessive mutation was identified in the GBSSI gene characterizing the inheritance of this phenotype [27]. However, this causal mutation determined by the deletion of a cytokine in exon 6 at position 1701 bp was not identified in the cassava germplasm analyzed here, so it will not be possible to explore the use of this mutation to generate segregating populations for the waxy gene in backgrounds other than the original source AM206–05 [34]. However, it is important to note that throughout the evolutionary process, different mutation points have already been described as responsible for the waxy phenotype in other amylaceous species. Therefore, other points of causal mutations can be discovered in cassava based on gene sequencing GBSSI in the whole cassava germplasm of Latin America. Indeed, unpublished results suggest there are at least two different mutations resulting in these waxy phenotypes in cassava (Ceballos, personal communication). In maize, several mutations related to waxy starch have been reported, including insertions and deletions of varying sizes as well as transposable elements and SNPs [15]. The most recent mutations include transposition of the rf2 gene [35], deletions in exons 7 and 10 [16] and transposable elements in exons 6 and 7 [21] identified as functional mutations in the GBSSI gene and related to the waxy phenotype in maize.

Another important aspect is the phenotyping of individuals for quantitative determination of the amylose content, which could allow the identification of alternative allelic patterns for different amylose contents. According to Dobo et al. [36], three polymorphism in rice: exon 1 (G/T polymorphism), exon 6 (A/C polymorphism), and exon 10 (C/T polymorphism), exhibited five different allelic patterns: TCC, TAC, GCC, GAC and GAT. The allelic forms TAC and TCC were found in low-amylose varieties, GCC in varieties with intermediate amylose content, and GAT and GAC in varieties with high levels of amylose.

In general, starch synthesis is complex and derived from the coordinated action of several enzymes and their isoforms [13]. An example is the Protein Targeting to Starch (PTST) identified in *Arabidopsis thaliana* (L.), which is related to amylose synthesis. This protein acts on targeting and GBSSI transport [37, 38], so amylose synthesis is not yet completely elucidated. Mutants without the PTST factor in *A. thaliana* do not produce amylose because the GBSS protein, which normally binds to the starch, cannot bind in the absence of PTST. Recently, the application of CRISPR-Cas9 was demonstrated to generate cassava clones with modified starch by targeted mutagenesis of *PTST1* genes. These cassava clones exhibited lower amylose content in comparison with the wild type, showing that PTST also participates in the synthesis of amylose in the cassava storage roots [26]. Therefore, it is possible that in addition to direct mutations at the gene level, other genomic regions may help to explain the waxy phenotype, and therefore be selectable.

The identification of different functional mutations in the PTST gene (Manes.02G075700) already described and mapped on cassava chromosome 2, as well as the other GBSSI isoform located on chromosome 1 (Manes.01G055700) [39], not evaluated in this work, may bring new contributions to a better understanding of the molecular bases of waxy gene expression in the germplasm of Latin America. Considering that the GBSSI gene is the only synthase known to be responsible for amylose synthesis and that the PTST protein is responsible for GBSSI targeting, a complete (paired-end) sequencing of such genes, including promoter regions, in the cassava germplasm would be one of the first steps in the search for alternative alleles for the waxy phenotype. All of the nucleotide diversity of the evaluated genes would allow an extensive in silico evaluation of points of potential mutations for inactivation of amylose production. This information, linked to the sequencing results, would direct the self-pollination of heterozygous accessions. With populations that have already been obtained, a molecular assessment of the segregation preceded by a field assessment using the iodine test would identify promising individuals. Finally, with validated markers in new sources of waxy genotypes, a KASP evaluation with different genetic backgrounds could be carried out, reaffirming the potential of these mutations to select potential cassava parents for waxy breeding.

## Conclusions

Previously reported GBSSI-related SNPs and those found in this work are not useful for molecular marker-assisted selection in genetic backgrounds other than the AM206–5 source. Only the deletion in exon 6 (MeWxEx6-del-C) was able to completely discriminate the non-waxy and waxy genotypes. Although the evaluated germplasm did not have the allele associated with the original AM206–5 source, our results contribute to the establishment of a practical model for the use of molecular-assisted selection for allelic variants associated with the waxy phenotype in cassava, with the goal of optimizing the genotyping and early identification of specific genotypes with the desired alleles for generation of segregating populations.

## Methods

### Screening of cassava germplasm with single nucleotide amplified polymorphism [SNAP] markers associated with the waxy phenotype in the source AM206–5

A total of 1529 accessions belonging to the Brazilian Cassava Germplasm Bank of Embrapa Mandioca e Fruticultura (“Brazilian Agricultural Research Corporation – Embrapa Cassava and Fruit”), originating from different ecosystems of Brazil, Colombia, Venezuela and Uganda, were analyzed (Supplement - Table S1). DNA extraction was performed according to the CTAB (cetyltrimethylammonium bromide) protocol [40]. To verify the quality and quantity of extracted DNA, ethidium bromide (1.0 mg. L^− 1^) was used to stain the DNA in 1% (w/v) agarose gel (Invitrogen, USA) and was visually compared with various concentrations of Lambda DNA (Invitrogen, USA).

The mutations located in exon 6 (deletion of 1 bp at nucleotide 92) and the SNP at exon 11 (GC to AT) found by Aiemnaka et al. [27] were not optimized even after several PCR adjustments. Therefore, the genotyping of cassava germplasm (landraces and improved varieties) was performed using only the single nucleotide amplified polymorphism (SNAP) primers MeWxI11-G and MeWxI11-C developed by Aiemnaka et al. [27] and located at intron 11 of the waxy gene (GBSSI). The polymerase chain reaction (PCR) reactions were performed in a final volume of 15 μL containing 10 ng of DNA, PCR 1x buffer, 1.5 mM of MgCl_2_ (4G, Brazil), 0.2 mM of dNTP (Promega, USA), 0.2 uM of each primer (Integrated DNA Technologies, USA), and 1 U of Taq DNA Polymerase (Promega, USA). The amplification program consisted of a cycle at 94 °C for 2 min; 30 cycles at 94 °C for 30 s; annealing steps at 56 °C for 30 s and 72 °C for 1 min; and a final extension at 72 °C for 5 min, performed using a Veriti® 96-well model thermocycler (Applied Biosystems, USA). For the development of the amplification products, 2% (w/v) 1000 agarose gel (Invitrogen, USA) containing 1.0 mg. L^− 1^ of ethidium bromide was used and electrophoresed in TBE 0.5x buffer (45 mM Tris-borate, 1 mM EDTA). The products on the gel were visualized in UV light and recorded with the Gel Logic 212 Pro Photodocumentator (Carestream Molecular Imaging, USA).

### Evaluation of S_1_ progenies using SNAP markers

Twenty-eight genotypes previously identified with the G allele at position 3197 bp of intron 11 (MeWxI11-G - associated with the waxy phenotype) in the heterozygous form [27] were self-fertilized for the generation of homozygous individuals with the waxy phenotype. The selected cassava accessions for self-pollination according to their flowering from August to November 2014 were BGM0061, BGM0131, BGM0132, BGM0222, BGM0463, BGM0505, BGM0614, BGM0650, BGM0726, BGM0729, BGM0741, BGM0872, BGM0935, BGM0941, BGM0962, BGM1023, BGM1041, BGM1120, BGM1143, BGM1148, BGM1253, BGM1284, BGM1288, BGM1335, BGM1378, BGM1383, BGM1413, and BGM1819.

To perform the self-pollination of the cassava accessions, the receptive female flowers were covered before opening in the morning with cloth bags (20 × 15 cm) to protect them from pollen contamination. Male flowers of the same genotype were collected in the morning and placed in pre-labeled, large-cap bottles. In the late morning and early afternoon, self-pollination was carried out through the contact of the anthers with the stigma of the female flower to ensure artificial pollination. Then, the pollinated flowers were covered with voile [light cotton cloth] until the collection of the seeds after natural dehiscence.

The seeds from the self-pollination of the 28 accessions were sown in a greenhouse, and after 30 days, the seedlings were transplanted to the field. The plants were harvested and evaluated 10 months after planting. The S_1_ plants of each family were evaluated for the presence of waxy starch using the 2% iodine test (2 g KI and 0.2 g I2 in distilled water), applied to a cross section of the roots of all genotypes [25]. The long amylose chains have a high ability to bind to the iodine in the solution, which gives a blue color to the starches containing amylose when stained with iodine. In contrast, amylopectin has a low iodine-binding capacity, and therefore, red-brown stains are characteristic of starches essentially containing only this polymer [7].

After the evaluations of S_1_ families in the field, 185 genotypes belonging to families S1-BGM0061 (60 individuals), S1-BGM0463 (24 samples), and S1-BGM0935 (101 samples) were genotyped with SNAP markers, as previously described. The molecular segregation for the GBSSI gene in the S_1_ individuals based on the primers MeWxI11-G and MeWxI11-C was evaluated by the χ^2^ test ${\sum}_{i=1}^n\frac{{\left[{f}_o-{f}_e\right]}^2}{f_e}$, where *f*_*o*_ and *f*_*e*_ are the observed and expected frequencies of the phenotypes.

### GBSSI gene sequencing

Complete GBSSI gene sequencing was performed on 89 cassava genotypes. Of this total, 54 accessions were considered homozygous (CC) or heterozygous (CG) using the primers MeWxI11-G and MeWxI11-C (non-waxy phenotype), and 35 genotypes were segregating from AM206–5 derived populations. Of the latter, 3 were homozygous (CC) or heterozygous (CG) (non-waxy phenotype), and 32 were homozygous (GG) genotypes (waxy phenotype) (Table 2). The extraction and quantification of the genomic DNA was performed as described previously.

**Table 2:** List of cassava accessions used in GBSSI (granule-bound starch synthase I) gene sequencing

**Non-waxy samples (*Wx_*)**				
9602–02	BGM0171	BGM0729	BGM1198	BGM1453
9607–07	BGM0222	BGM0741	BGM1287	BGM1594
BGM0023	BGM0263	BGM0752	BGM1288	BGM1596
BGM0043	BGM0326	BGM0756	BGM1328	BGM1598
BGM0046	BGM0336	BGM0776	BGM1335	BGM1692
BGM0087	BGM0360	BGM0785	BGM1339	BGM1711
BGM0116	BGM0362	BGM0841	BGM1342	7734–03
BGM0132	BGM0378	BGM1118	BGM1354	7734–05
BGM0145	BGM0399	BGM1140	BGM1364	7745–01
BGM0146	BGM0536	BGM1143	BGM1444	
BGM0149	BGM0545	BGM1144	BGM1448	
BGM0170	BGM0592	BGM1161	BGM1451	
**Waxy samples (*wxwx*)**				
6383–01	7690–08	7734–06	7737–07	7747–01
6413–01	7694–03	7734–07	7737–08	8109–03
6421–05	7697–03	7736–05	7738–01	8109–04
6428–01	7698–14	7736–07	7745–02	8109–05
6430–01	7698–16	7737–01	7745–04	
6437–01	7734–01	7737–02	7745–05	
6477–02	7734–02	7737–06	7745–06	

For complete amplification of the gene, five primers were developed using the reference sequence of the GBSSI gene (Manes.02G001000) from cassava genome v6.1 [39] deposited in the Phytozome v12.1 database [41] (Table 3). The Primer3 program [42] was used to design primers with the following criteria: product size amplified between 800 and 1100 bp; annealing temperature above 60 °C; and G / C percentage above 40%. The primers were allocated to overlap each of the sequences to ensure complete gene coverage.

**Table 3:** Primers used for amplification and complete sequencing of the GBSSI (granule-bound starch synthase I) gene in waxy and non-waxy cassava genotypes

Primer	Sequence	Expected fragment size (bp)
MeGBSSIpA-F	TGGCGAAGTCCCACCATTAC	974
MeGBSSIpA-R	TGTACTGGTCATAGCGGGGA	
MeGBSSIpB-F	TCCCCGCTATGACCAGTACA	988
MeGBSSIpB-R	ACAAGTCACCAACCCCGAAA	
MeGBSSIpC-F	CACTGCTCTGCTTCCATGTTATCT	936
MeGBSSIpC-R	TCTCACAACACAACCAAGGACATC	
MeGBSSIpD-F	CAGAAGTCGGATTGCCTGTTGATA	931
MeGBSSIpD-R	GCTACCAGTCAATCCAATTTGCAC	
MeGBSSIpE-F	TGACTAAGTATCTAGGAGGCTCA	1038
MeGBSSIpE-R	GAAGGGAAGAAAGAAACTGAATGAC	

For primers optimization, different annealing temperatures (58 to 64 °C), magnesium chloride concentrations (1, 1.5 and 2 mM), and different numbers of PCR extension cycles were tested. PCR reactions were optimized in final volume of 50 μl containing 10 ng of DNA, 1X PCR buffer, 1.5 / 2.0 mM MgCl 2 (Invitrogen, USA), 0.2 mM dNTP (Promega, USA), 2 mM of each primer (Integrated DNA Technologies, USA), and 1 U of Taq DNA High Fidelity Polymerase (Invitrogen, USA). The amplification program was optimized using an initial denaturation of one cycle at 95 °C for 1 min; followed by 30/35 cycles at 95 °C for 15 s, annealing at 62 °C for 15 s and 72 °C for 30 s; and final extension at 72 °C for 7 min, performed in a Veriti® 96-well thermocycler (Applied Biosystems, USA).

The electrophoresis product was purified with ExoSap-IT (Affymetrix, USA), sequenced in both directions using BigDye® Terminator v3.1 Cycle Sequencing (Applied Biosystems, USA) and analyzed with an ABI Prism 3730 XL analyzer (Applied Biosystems, USA). The chromatograms were assigned and trimmed with Phred [43, 44], aligned with the reference sequence Manes.02G001000, and the SNPs were identified with the aid of the newSNP v3.0.1 program [45]. Identification of the coding and non-coding regions was performed by comparison with the GBSSI gene (Manes.02G001000) of the cassava genome v6.1 [39].

### Discriminant analysis of principal components

Discriminant analysis of principal components (DAPC) was performed based on the allelic variants identified by the complete GBSSI gene sequencing. DAPC was performed, retaining more than 90% of the variation of the data with the first 10 principal components and one discriminant function. The analysis was performed using the *adegenet* package [46] of the software R v3.5 [47].

### Genotypic evaluation of the cassava germplasm for deletion MeWxEx6-del-C

The Kompetitive Allele Specific PCR (KASP) technology [28] for screening the deletion MeWxEx6-del-C was performed by the company Intertek AgriTech. The KASP genotyping optimization was performed in two stages. In the first step, a set of individuals with known allelic conditions was used, i.e., 31 individuals homozygous for the waxy gene (*wxwx*); 31 heterozygous individuals (*Wxwx*) from segregating populations for the waxy genotype derived from the AM206–5 source, and 32 non-waxy individuals (*WxWx*) from the germplasm bank (Table 4). Allele-specific fluorescence (HEX and FAM) was detected using the SNPviewer2 v4.0.0 program. and clusters were visualized using R v.3.5 [47]. After optimization and validation of the MeWxEx6-del-C marker, genotyping of 1529 cassava accessions was performed.

**Table 4:** Cassava genotypes used to optimize the genotyping of the cytosine deletion at exon 6, position 1701 bp (MeWxEx6-del-C) by Kompetitive Allele Specific PCR (KASP)

**Homozygous waxy samples (*wxwx*)**				
6460–2	7474–1	7799–2	7909–6	8034–2
6466–3	7738–4	7802–3	7921–1	8093–3
6502–1	7745–5	7807–5	7934–1	8109–4
6703–1	7751–1	7811–6	7950–3	
6896–4	7754–3	7813–1	7953–2	
7020–1	7773–6	7867–4	7992–3	
7429–3	7788–7	7882–2	8014–7	
**Heterozygous non-waxy samples (*Wxwx*)**				
2017wx-01-01	2017wx-02-12	2017wx-02-43	2017wx-03-09	2017wx-03-29
2017wx-01-02	2017wx-02-17	2017wx-03-01	2017wx-03-16	2017wx-03-31
2017wx-01-03	2017wx-02-18	2017wx-03-02	2017wx-03-18	2017wx-03-38
2017wx-01-06	2017wx-02-19	2017wx-03-03	2017wx-03-20	
2017wx-01-07	2017wx-02-25	2017wx-03-06	2017wx-03-21	
2017wx-02-10	2017wx-02-26	2017wx-03-07	2017wx-03-22	
2017wx-02-11	2017wx-02-36	2017wx-03-08	2017wx-03-28	
**Homozygous non-waxy samples (*WxWx*)**				
032–09	BGM2326	Conquista 1	JoselitoA2	Roxona
517–08	BGM2327	Conquista 2	Ouro Pão	RR0065
Aciolina	BGM2333	CS01	Peru Preto	Tailandesa
Aipim Abacate	BRS396	Folha Fina	Pretinha	Venâncio-RN
Amarelona	BRS399	Inajazinha	Rl-F	
AM-Jaeve-RN	CL-Acre	Ipirá	Retori	
BGM0001	CL-Rl	Jacona	Roxinha	

## Supplementary information

Additional file 1: **Figure S1.** Example of amplification of the primers MeWxI11-G and MeWxI11-C in 2% agarose gel stained with ethidium bromide in the cassava populations S_1_-BGM0061. HT - Heterozygous (CG), HD - Dominant Homozygous (CC), and HR - Homozygous recessive (GG).

Additional file 2: **Table S1.** List of cassava clones used in screening with single nucleotide amplified polymorphism [SNAP] markers.

## Data Availability

The datasets used and/or analysed during the current study are available from the corresponding author on reasonable request.

## Abbreviations

GBSSI: Granule-bound starch synthase I (GBSSI) DAPC Discriminant principal component analysis
KASP: Kompetitive Allele Specific PCR
SNP: Single-nucleotide polymorphism
MeWxI11-G/C: Single nucleotide polymorphisms in intron 11 indicated as G/C base substitution
MeWxEx6-del-C: 1-bp deletion at nucleotide 92 of exon 6 that creates a premature stop codon on GBBSI gene
MAS: Molecular-assisted selection
SNAP: Single nucleotide-amplified polymorphism markers
UTR: Untranslated region
