# Supplementary figures and images for: Identification and validation of mutation points associated with waxy phenotype in cassava
Source: BMC Plant Biol. 2020 Apr 15;20:164. doi: 10.1186/s12870-020-02379-3 (PMC7160975; doi:10.1186/s12870-020-02379-3)

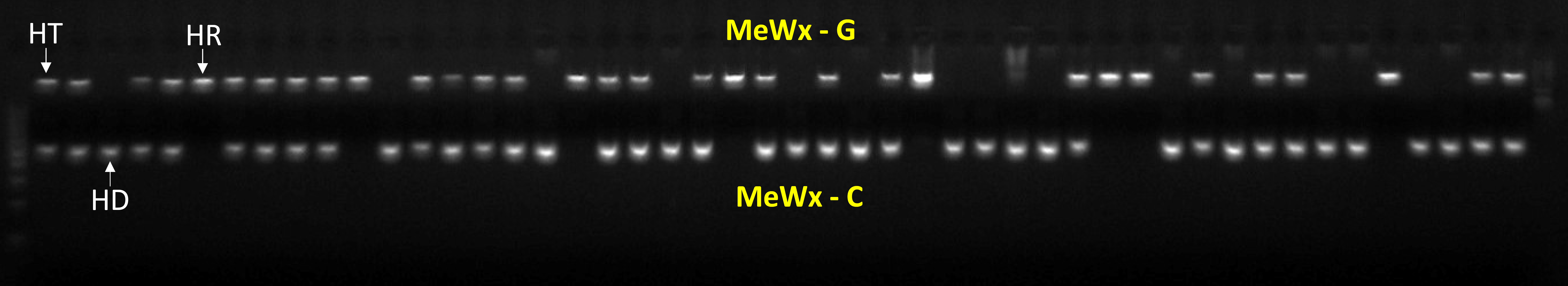

Supplement: Supplementary file 1 — Figure S1. Example of amplification of the primers MeWxI11-G and MeWxI11-C in 2% agarose gel stained with ethidium bromide in the cassava populations S1-BGM0061. HT - Heterozygous (CG), HD - Dominant Homozygous (CC), and HR - Homozygous recessive (GG). [file 12870_2020_2379_MOESM1_ESM.tif]
